# The influence of mode of anaesthesia on perioperative outcomes in people with hip fracture: a prospective cohort study from the National Hip Fracture Database for England, Wales and Northern Ireland

**DOI:** 10.1186/s12916-022-02517-8

**Published:** 2022-09-26

**Authors:** Gulraj S. Matharu, Anjali Shah, Samuel Hawley, Antony Johansen, Dominic Inman, Iain Moppett, Michael R. Whitehouse, Andrew Judge

**Affiliations:** 1 Musculoskeletal Research Unit, Bristol Medical School, University of Bristol, Level 1 Learning and Research Building, Southmead Hospital, Westbury-on-Trym, Bristol, BS10 5NB UK; 2 Nuffield Department of Orthopaedics, Rheumatology and Musculoskeletal Sciences, University of Oxford, Nuffield Orthopaedic Centre, Oxford, OX3 7LD UK; 3 University Hospital of Wales and School of Medicine, Cardiff University, Cardiff, UK; 4 Department of Orthopaedics, Northumbria Healthcare NHS Foundation Trust, North Shields, UK; 5 Anaesthesia and Critical Care Section Academic Unit of Injury, Recovery and Inflammation Sciences, Queen’s Medical Centre, University of Nottingham, Nottingham, UK; 6 National Institute for Health Research Bristol Biomedical Research Centre, Bristol, UK

**Keywords:** Anaesthesia, Spinal, General, Outcomes, Hip fracture, Delirium

## Abstract

**Background:**

Delirium is common after hip fracture surgery, affecting up to 50% of patients. The incidence of delirium may be influenced by mode and conduct of anaesthesia. We examined the effect of spinal anaesthesia (with and without sedation) compared with general anaesthesia on early outcomes following hip fracture surgery, including delirium.

**Methods:**

We used prospective data on 107,028 patients (2018 to 2019) from the National Hip Fracture Database, which records all hip fractures in patients aged 60 years and over in England, Wales and Northern Ireland. Patients were grouped by anaesthesia: general (58,727; 55%), spinal without sedation (31,484; 29%), and spinal with sedation (16,817; 16%). Outcomes (4AT score on post-operative delirium screening; mobilisation day one post-operatively; length of hospital stay; discharge destination; 30-day mortality) were compared between anaesthetic groups using multivariable logistic and linear regression models.

**Results:**

Compared with general anaesthesia, spinal anaesthesia without sedation (but not spinal with sedation) was associated with a significantly reduced risk of delirium (odds ratio (OR)=0.95, 95% confidence interval (CI)=0.92–0.98), increased likelihood of day one mobilisation (OR=1.06, CI=1.02–1.10) and return to original residence (OR=1.04, CI=1.00–1.07). Spinal without sedation (*p*<0.001) and spinal with sedation (*p*=0.001) were both associated with shorter hospital stays compared with general anaesthesia. No differences in mortality were observed between anaesthetic groups.

**Conclusions:**

Spinal and general anaesthesia achieve similar outcomes for patients with hip fracture. However, this equivalence appears to reflect improved perioperative outcomes (including a reduced risk of delirium, increased likelihood of mobilisation day one post-operatively, shorter length of hospital stay and improved likelihood of returning to previous residence on discharge) among the sub-set of patients who received spinal anaesthesia without sedation. The role and effect of sedation should be studied in future trials of hip fracture patients undergoing spinal anaesthesia.

**Supplementary Information:**

The online version contains supplementary material available at 10.1186/s12916-022-02517-8.

## Background

Over 70,000 hip fractures occur each year in the United Kingdom (UK) and almost all receive urgent surgery [1]. These often frail, older patients face substantial morbidity; 6–10% die within 1 month [1] or experience reduced health-related quality of life [2]. The commonest post-operative complication is delirium, often under-detected despite its deleterious effect on patient experience and recovery [3–5]. Delirium occurs in between a quarter and half of patients [1, 6, 7]. People with hip fractures commonly require prolonged admissions, are often less mobile than pre-operatively and need more care post-discharge. Patients recovering from hip fracture occupy over 4000 hospital beds in the UK [8], with annual hospital costs of £1.1 billion (1% of the National Health Service (NHS) budget) [9].

Surgery can be performed under general or regional (usually spinal) anaesthesia. A Cochrane analysis of 28 randomised controlled trials (RCTs) (*n*=2976) [10], highlighted low-quality evidence and no difference between general or spinal anaesthesia for 30-day mortality, pneumonia, myocardial infarction, cerebrovascular accident, and acute confusional state [10]. Trials were noted to not reflect current practice; patients were often sedated before spinal anaesthesia but this was not considered in analyses. Observational studies report contrasting findings regarding mortality, readmissions, complications, and length of stay [11–17]. Most studies have limited detail on anaesthesia technique and co-interventions (e.g. sedation), and focus on outcomes 30 days or more after surgery, rather than on important, distressing short-term outcomes like post-operative delirium. The recent Regional versus General Anesthesia for Promoting Independence after Hip Fracture (REGAIN) study [18] found no difference in outcomes, including delirium, when patients with hip fracture were randomised to general or spinal anaesthesia, but the effects of sedation were not examined within this RCT.

The National Institute for Health and Care Excellence (NICE) have made a high-priority recommendation for a three-arm RCT to compare general anaesthesia versus spinal anaesthesia without sedation versus spinal anaesthesia with sedation on postoperative outcomes after hip fracture [19]. In addition, the fragility fractures James Lind Alliance priority setting partnership, undertaken by patients and healthcare professionals, highlighted two important research questions for hip fracture patients (identifying the optimal pain relief during anaesthesia and post-operatively; and finding the best treatments to prevent/treat delirium post-operatively) [20, 21].

The National Hip Fracture Database (NHFD) for England, Wales and Northern Ireland is a mandatory national clinical audit of hip fracture care, with hospitals continually assessed against Key Performance Indicators (KPIs) [1]. These include two measures of acute peri-operative care: success in getting patients out of bed by the day after surgery and post-operative delirium assessment. These important short-term outcomes are missing from previous observational data and RCTs [10], including recent trials [22, 23]. Delirium is a common complication of surgery in frail and older people, distressing to patients, family and carers, and associated with increased mortality or institutional care placement. The 4 ‘A’s Test (4AT) is a rapid delirium screen, in patients with or without cognitive impairment, and can predict immobility, prolonged length of stay, mortality and change in residence on discharge [6].

The NHFD dataset includes details of casemix including an admission cognitive assessment (Abbreviated Mental Test Score (AMTS)) and details of the care, fracture type, surgery and anaesthesia. We hypothesised that mode of anaesthesia would be associated with risk of post-operative delirium. We used the NHFD to assess the effect of spinal anaesthesia (with and without sedation) and general anaesthesia on early postoperative outcomes, including delirium and mobilisation by the day after surgery, and other relevant postoperative outcomes including length of stay, discharge destination and 30-day mortality.

## Methods

### Study design and data sources

A prospective cohort study was performed using NHFD data. It contains data on over 97% of all hip fractures in patients aged 60 years or above in England, Wales and Northern Ireland [1]. These include patient characteristics, hip fracture type, surgery, details of the care patients receive and relevant outcomes. Data are collected and submitted by clinical teams in 175 trauma units. Patients’ details and NHS number are passed to the NHS Personal Demographics Service, which provided the date of death from the Office for National Statistics (ONS).

The Healthcare Quality Improvement Partnership (HQIP) is commissioned by NHS England to commission and manage the National Clinical Audit and Patient Outcomes Programme (NCAPOP). As part of this programme, the NHFD is a quality improvement initiative commissioned by HQIP/NHS England. NHFD data is collected under section 251 of the NHS Act 2016 following approval by the Health Research Authority (HRA) Confidentiality Advisory Group (CAG 8-03(PR11)/2013). Only pseudonymised data are sent to the University of Oxford for this project. This research project was reviewed by HQIP and approved as an extended analysis and output of the NHFD clinical audit programme. Ethical approval was not sought in line with Governance Arrangements for Research Ethics Committee (GAfREC) guidance for this secondary analysis of administrative data.

### Study selection criteria

**Fig. 1:**
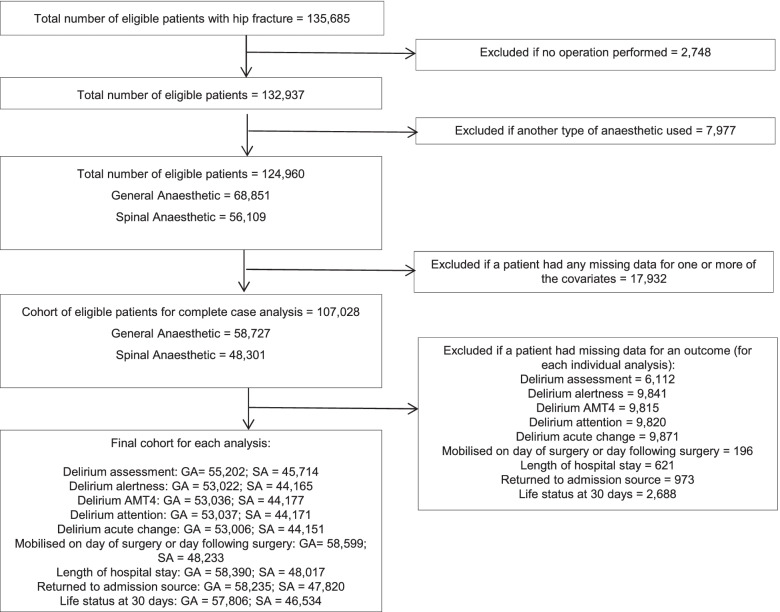
Study selection criteria using data from the National Hip Fracture Database for England, Wales and Northern Ireland during 2018–2019

We included patients who presented over a 2-year period (1st January 2018 to 31st December 2019; *n*=135,685). The following exclusions were made: (1) patients who did not undergo surgery (*n*=2748); (2) patients who received anaesthesia other than the defined exposures (see below; *n*=7977); (3) patients with missing data for one or more of the covariates (*n*=17,932). After these exclusions, there were 107,028 patients (58,727 (55%) general anaesthesia and 48,301 (45%) spinal anaesthesia) available for the complete case analysis (Fig. 1). For each individual analysis of the outcomes of interest, patients were excluded if they did not have data available for that outcome (Fig. 1).

### Exposure

The NHFD collects data on anaesthesia type, including the use of sedation and nerve blocks. The data available for both of these variables are binary; for example, the patient either did or did not receive sedation. The primary anaesthetic exposure of interest was binary: general or spinal anaesthesia (with or without sedation). Patients who received both general and spinal anaesthesia were excluded, as were any who received epidural anaesthesia [24]. These primary anaesthetic groupings made no distinction regarding the use of nerve blocks (which were adjusted for in the statistical models).

### Covariates

Potential confounding factors for the outcomes being assessed were chosen a priori to be adjusted for in the subsequent analyses [8, 11, 25–28]. These covariates were patient age at surgery, sex, American Society of Anesthesiologists (ASA) physical status, year of presentation to hospital, fracture type, pathological fracture or not, hospital geographical region, pre-injury residence from which patient was admitted, cognitive state (admission AMTS), pre-injury mobility, whether the patient received a nerve block in the Emergency Department or on the ward pre-operatively, time from admission to theatre, grade of senior surgeon in theatre, grade of senior anaesthetist present in theatre, the use of a nerve block in theatre, and operation performed. The NHFD uses ASA physical status rather than a measure specifically focused on frailty. The ASA physical status ranges from 1 (healthy patient) to 5 (moribund patient not expected to live for more than 24 h with or without surgery) [29]. AMTS is scored from 0 to 10 and grouped according to clinically relevant classifications (0 to 7 represents abnormal cognition, and 8 to 10 represents normal cognition) [30].

### Outcomes

Outcomes of interest included postoperative delirium in the week after surgery, mobilisation by the day after surgery, length of acute hospital stay, discharge destination, and 30-day mortality. The result of a delirium assessment performed using the 4AT score [31] in the week after surgery is a KPI for the NHFD. The 4AT score ranges from 0 to 12; a score of 0 suggests unlikely delirium or cognitive impairment; 1 to 3 suggests possible chronic cognitive impairment without excluding the possibility of delirium; 4 or more suggests delirium with or without chronic cognitive impairment [6, 31].

The 4AT score is calculated from the summation of its 4 subscales: alertness (scored 0=normal, with mild sleepiness for <10 seconds after waking, or 4=abnormal), Abbreviated Mental Test 4 (AMT4) (0=no mistakes; 1=one mistake; 2=two or more mistakes or untestable), attention (0=reciting ≥7 months backwards correctly; 1=starts but lists <7 months or refuses to start; 2=untestable), and acute change or fluctuating course (0=no or 4=yes). The AMT4 tests for recall of age, date of birth, place (name of hospital or building) and current year. Attention is tested by instructing the patient to list months in reverse order, starting from December. Acute change or fluctuating course is the evidence of significant change or fluctuation in mental status within the last 2 weeks and persisting in the last 24 h.

### Statistical analysis

Analyses were conducted using STATA version 15.1 (StataCorp, TX, USA), with 95% confidence intervals (CIs). Descriptive statistics were used to summarise patients’ demographic and clinical factors stratified by the anaesthesia exposure variable. The standardised mean difference (SMD) for each covariate by anaesthetic type was used to measure covariate imbalance. SMDs of 0.10 or more for any covariate were suggestive of imbalance [32, 33], with only one covariate (pre-operative cognitive state) having evidence of imbalance (Table 1).

**Table 1:** Baseline characteristics of hip fracture patients treated in England, Wales and Northern Ireland during 2018–2019, as recorded in the National Hip Fracture Database

		Total		General anaesthetic		Spinal anaesthetic		Standardised mean difference
		Number	%	Number	%	Number	%	
**Total**		124,960	100	68,851	55	56,109	45	
**Year**	2018	61,869	50	33,603	49	28,266	50	− 0.029
	2019	63,091	50	35,248	51	27,843	50	
**Age** (continuous)^a^		Mean=82.7 SD=8.6		Mean=82.7 SD=8.7		Mean=82.6 SD=8.6		− 0.012
**Age groups**	60–69 years	10,937	9	6161	9	4776	9	
	70–79 years	29,250	23	15,733	23	13,517	24	
	80–89 years	56,469	45	31,299	45	25,170	45	
	90+ years	28,304	23	15,658	23	12,646	23	
**Sex**	Female	88,436	71	48,807	71	39,629	71	0.007
	Male	36,524	29	20,044	29	16,480	29	
**Fracture type**	Intracapsular	72,704	58	38,562	56	34,142	61	− 0.098
	Extracapsular, including other	52,137	42	30,196	44	21,941	39	
	Missing	119	<1	93	<1	26	<1	
**Pathology**	No malignancy	119,033	95	65,473	95	53,560	95	− 0.006
	Malignancy present	1478	1	833	1	645	1	
	Missing	4449	4	2545	4	1904	3	
**Region**	East Midlands	8808	7	4241	6	4567	8	0.035
	East of England	13,887	11	8777	13	5110	9	
	London	10,926	9	6379	9	4547	8	
	North East	6887	6	3403	5	3484	6	
	North West	15,897	13	9045	13	6852	12	
	Northern Ireland	4009	3	1322	2	2687	5	
	South Central	8101	6	4885	7	3216	6	
	South East	10,812	9	4588	7	6224	11	
	South West	14,058	11	8,683	13	5,375	10	
	Wales	7691	6	4657	7	3,034	5	
	West Midlands	12,076	10	7603	11	4,473	8	
	Yorkshire and the Humber	11,808	9	5268	8	6540	12	
**Admission source**	Own home/sheltered housing	102,292	82	54,868	80	47,424	85	− 0.031
	Nursing care	8994	7	5457	8	3537	6	
	Residential care	13,611	11	8488	12	5123	9	
	Missing	63	<1	38	<1	25	<1	
**ASA physical status**	ASA 1 or 2	28,940	23	14,961	22	13,979	25	− 0.093
	ASA 3	71,734	57	39,666	58	32,068	57	
	ASA 4 or 5	22,341	18	12,979	19	9362	17	
	Missing	1945	2	1245	2	700	1	
**Preinjury mobility**	Freely mobile without aids	45,388	36	23,970	35	21,418	38	− 0.097
	Mobile outdoors with one aid or two aids or frame	46,228	37	25,326	37	20,902	37	
	Some indoor mobility but never goes out, or no functional mobility	32,207	26	18,827	27	13,380	24	
	Missing	1137	1	728	1	409	1	
**Pre-operative cognitive state (Abbreviated Mental Test Score)**	0–7	43,690	35	26,202	38	17,488	31	0.15
	8–10	75,834	61	39,738	58	36,096	64	
	Missing	5436	4	2911	4	2525	5	
**Operation type**	Hemiarthroplasty	55,176	44	29,661	43	25,515	45	0.07
	Total hip replacement	10,030	8	4709	7	5321	9	
	Internal fixation—cannulated screws	3291	3	1817	3	1474	3	
	Internal fixation—intramedullary nail	17,783	14	10,929	16	6854	12	
	Internal fixation—sliding hip screw	38,402	31	21,564	31	16,838	30	
	Other/missing	278	<1	171	<1	107	<1	
**Grade of surgeon**	Consultant	87,934	70	48,032	70	39,902	71	− 0.022
	Other	36,714	29	20,617	30	16,097	29	
	Missing	312	<1	202	<1	110	<1	
**Grade of anaesthetist**	Consultant	106,803	85	59,107	86	47,696	85	0.039
	Other	16,712	13	8805	13	7863	14	
	Missing	1444	1	894	1	550	1	
**Time to theatre from admission**^b^		Median=24.7 IQR=18.7–40.6		Median=24.7 IQR=18.7–40.4		Median=24.7 IQR=18.7–40.9		0.008
**Time to theatre from admission ≥36 hours**	Yes	37,908	30	20,734	30	17,174	31	0.008
	No	87,052	70	48,117	70	38,935	69	
**Nerve block in the Emergency Department or the ward before arrival in theatre suite**	Yes	60,848	49	32,713	48	28,135	50	0.058
	No	58,930	47	33,295	48	25,635	46	
	Missing	5182	4	2843	4	2339	4	

^a^*SD* standard deviation

^b^*IQR* interquartile range

Multivariable regression models were used to assess the effect of anaesthesia on each outcome of interest. All regression models were a priori adjusted for the covariates described above, including admission AMTS, with analyses conducted using a complete case analysis. For delirium, analyses were performed using the 4AT score (grouped 0, 1 to 3, and 4 or more). Additional file 1: Appendix 1 details the types of regression models used for each outcome, tests of assumptions and sensitivity analysis.

## Results

### General anaesthesia versus spinal anaesthesia (Tables 2 and 3)

In the adjusted regression models, use of spinal anaesthesia was associated with a significantly reduced odds of delirium (4AT), when compared with general anaesthesia (odds ratio (OR)=0.96, 95% CI=0.94–0.99; *p*=0.007). More patients returned to their original residence with spinal anaesthesia (OR=1.04, CI=1.01–1.06; *p*=0.013), which also had a significantly shorter length of hospital stay (coefficient −0.46 days, CI= −0.66 to −0.26 days; *p*<0.001). There were no differences in mobilisation by the day after surgery (OR=1.02, CI=0.99–1.06; *p*=0.156), or in 30-day mortality (OR=1.03, CI=0.98–1.09; *p*=0.248).

**Table 2:** Outcomes of hip fracture patients after surgery by anaesthetic type for those treated in England, Wales and Northern Ireland during 2018–2019, as recorded in the National Hip Fracture Database

Outcome		Total		General anaesthetic		Spinal anaesthetic	
		Number	%	Number	%	Number	%
**Total**		124,960	100	68,851	55	56,109	45
**Delirium assessment**	0 - Delirium or cognitive impairment unlikely	55,410	44	28,888	42	26,522	47
	1-3 Possible cognitive impairment	27,232	22	15,055	22	12,177	22
	4+ Possible delirium or cognitive impairment	32,244	26	19,036	28	13,208	24
	Missing	10,074	8	5872	9	4202	7
**Delirium alertness**	0	100,452	80	54,490	79	45,962	82
	4	7029	6	4294	6	2735	5
	Missing	17,479	14	10,067	15	7412	13
**Delirium AMT4**	0	62,233	50	32,351	47	29,882	53
	1	13,188	11	7110	10	6078	11
	2	32,119	26	19,363	28	12,756	23
	Missing	17,420	14	10,027	15	7393	13
**Delirium attention**	0	61,788	49	32,290	47	29,498	53
	1	21,361	17	11,978	17	9383	17
	2	24,386	20	14,558	21	9828	18
	Missing	17,425	14	10,025	15	7400	13
**Delirium acute change**	0	95,723	77	51,978	75	43,723	78
	4	4960	4	6795	10	4960	9
	Missing	7426	6	10,078	15	7426	13
**Mobilised on day of or day following surgery**	No	24,752	20	14,071	20	10,681	19
	Yes	99,772	80	54,502	79	45,270	81
	Missing	436	<1	278	<1	158	<1
**Length of hospital stay (days)**^a^		Median=15 IQR=9–25		Median=15 IQR=9–26		Median=15 IQR=9–24	
**Returned to admission source**	Yes	81,060	65	44,128	64	36,932	66
	No	42,463	34	23,918	35	18,545	33
	Missing	1437	1	805	1	632	1
**Life status at 30 days**	Alive	117,998	94	64,824	94	53,174	95
	Dead	6962	6	4027	6	2935	5

^a^*IQR* interquartile range

**Table 3:** Multivariable regression analysis results for the effect of anaesthetic type (general anaesthetic vs. spinal anaesthetic) on outcome following surgery for hip fracture patients in England, Wales and Northern Ireland during 2018-19, as recorded in the National Hip Fracture Database

Outcome of interest	Adjustment for all variables		
	Odds ratio^a^	95% confidence interval	***P*** value
Delirium assessment (categorical)	0.96	0.94–0.99	0.007
Delirium alertness	0.90	0.86–0.96	<0.001
Delirium AMT4 1 vs 0	1.00	0.95–1.04	0.859
Delirium AMT4 2 vs 0	0.89	0.85–0.93	<0.001
Delirium attention	1.00	0.97–1.03	0.969
Delirium acute change	0.99	0.94–1.03	0.496
Mobilised on day of or day following surgery	1.02	0.99–1.06	0.156
Length of hospital stay	Linear regression coefficient = −0.46	−0.66 to −0.26	<0.001
Returned to admission source	1.04	1.01–1.06	0.013
Life status at 30 days	1.03	0.98–1.09	0.248

^a^General anaesthetic was the reference group

### General anaesthesia versus spinal anaesthesia without sedation versus spinal anaesthesia with sedation

The above adjusted regression analyses were repeated with the spinal anaesthesia group subdivided into those who did and did not receive sedation; 58,727 (55%) received general anaesthesia, 31,484 (29%) received spinal without sedation, and 16,817 (16%) received spinal with sedation. Baseline characteristics (Additional file 2: Table S1) and outcomes (Additional file 2: Table S2) for these three different anaesthetic exposure groups are provided. In most cases, the beneficial clinical effects associated with spinal anaesthesia (compared with general anaesthesia) in the previous analyses were only seen in the sub-group of patients who received spinal anaesthesia without sedation (Table 4).

**Table 4:** Multivariable regression analysis results for the effect of anaesthetic type (general anaesthetic vs. spinal anaesthetic with no sedation vs. spinal anaesthetic with sedation) on outcome following surgery for hip fracture patients in England, Wales and Northern Ireland during 2018–2019, as recorded in the National Hip Fracture Database

Outcome of interest		Adjustment for all variables		
		Odds ratio*	95% confidence interval	***P*** value
Delirium assessment (categorical)	Spinal anaesthetic only	0.95	0.92–0.98	0.001
	Spinal anaesthetic with sedation	1.00	0.96–1.04	0.854
Delirium Alertness	Spinal anaesthetic only	0.88	0.83–0.94	<0.001
	Spinal anaesthetic with sedation	0.94	0.87–1.02	0.165
Delirium AMT4	Spinal anaesthetic only	0.91	0.87–0.94	<0.001
	Spinal anaesthetic with sedation	0.94	0.89–0.98	0.008
Delirium Attention	Spinal anaesthetic only	0.97	0.94–1.00	0.084
	Spinal anaesthetic with sedation	1.06	1.01–1.11	0.009
Delirium Acute change	Spinal anaesthetic only	0.98	0.93–1.03	0.442
	Spinal anaesthetic with sedation	0.99	0.93–1.06	0.832
Mobilised on day of or day following surgery	Spinal anaesthetic only	1.06	1.02–1.10	0.004
	Spinal anaesthetic with sedation	0.96	0.92–1.01	0.117
Length of hospital stay	Spinal anaesthetic only	Linear regression coefficient = −0.45	−0.68 to −0.23	<0.001
	Spinal anaesthetic with sedation	Linear regression coefficient = −0.48	−0.77 to −0.19	0.001
Returned to admission source	Spinal anaesthetic only	1.04	1.00–1.07	0.025
	Spinal anaesthetic with sedation	1.03	0.99–1.08	0.106
Life status at 30 days	Spinal anaesthetic only	1.06	0.99–1.12	0.095
	Spinal anaesthetic with sedation	0.99	0.91–1.08	0.773

*General anaesthetic was the reference group

Compared with general anaesthesia, spinal without sedation (but not spinal with sedation) was associated with a 5% reduced odds of delirium (OR=0.95, CI=0.92–0.98; *p*=0.001), a 6% increased odds of mobilisation by the day after surgery (OR=1.06, CI=1.02–1.10; *p*=0.004), a 4% increased odds of return to original residence (OR=1.04, CI=1.00–1.07; *p*=0.025), and a significantly shorter length of hospital stay (without sedation coefficient −0.45 days, CI= −0.68 to −0.23 days, *p*<0.001; with sedation coefficient −0.48 days, CI= −0.77 to −0.19 days, *p*=0.001). To illustrate the differences observed in length of hospital stay, if the 58,727 patients receiving general anaesthesia had actually received spinal anaesthesia without sedation, this may have potentially reduced the total length of hospital stay for these patients by 26,427 days (CI 13,507 to 39,934 days), which we would consider to be of clinical importance. There were no differences observed in 30-day mortality between the three groups. Additional file 1: Appendix 1 provides more information on the results of the sensitivity analysis.

## Discussion

Whether mode of anaesthesia has a causal role in differences in outcomes following hip fracture surgery remains controversial. A Cochrane review reported no difference between general or spinal anaesthesia, but emphasised the available evidence was low quality with trials not reflecting current clinical practice [10]. Large observational studies have been limited by the heterogeneous nature of the spinal anaesthesia group, and have focussed on outcomes 30 days or more after surgery [11–17]. Such time frames for outcome assessment when assessing the effect of anaesthesia are no longer considered relevant by national clinical bodies, with a preference for more immediate, temporally related, and plausible perioperative outcomes such as post-operative delirium [34].

Delirium is the commonest complication of hip fracture and is associated with increased mortality, morbidity and healthcare costs [1, 6, 7]. Despite this it is often under-recognised. Little is known about how anaesthesia effects delirium especially in the short-term [10], although a recently completed trial in China is using delirium within 7 days of hip fracture surgery as the primary outcome and will help address this [35]. The recent REGAIN trial provides powerful reassurance that the choice of general or spinal anaesthesia is something that anaesthetists and their patients can safely decide between themselves, since neither approach had significantly better outcomes, including risk of post-operative delirium [18].

Our work has demonstrated that compared with general anaesthesia, the use of spinal anaesthesia without sedation was associated with a significantly reduced risk of delirium reflecting improved scores in the 4AT domains of ‘Alertness’ and AMT4 (four items testing orientation in time and place) [31], but not those of ‘Attention’, and ‘Acute change’. The observed delirium risk was reduced by 5% (CI=2% to 8%) with spinal without sedation (compared with general anaesthesia), which we consider to be of clinical relevance given that only 30% of patients currently receive spinal without sedation in this frail population, and especially given the known morbidity associated with delirium. Our work therefore suggests that spinal without sedation may therefore be the regimen most suitable for patients with hip fracture. Both a Cochrane review [10] and NICE [19] have previously postulated that the use of sedation with spinal anaesthesia may affect outcomes after hip fracture surgery. It was noted that previous trials often included patients sedated before spinal anaesthesia, which might reduce any short-term benefits [10].

Recent work demonstrated that the 4AT predicts a number of adverse outcomes following hip fracture surgery, including immobility, prolonged length of hospital stay, and change in residence on discharge [6]. The improvements seen for these outcomes in the spinal without sedation group of our study may reflect the reduced risk of delirium that we observed in these patients. These clinical benefits should not be underestimated in the frail and vulnerable hip fracture patient population. Immobility is associated with significant morbidity and often mortality, including pulmonary and urine sepsis, pressure ulcers, and venous thromboembolism [36, 37], therefore prompt mobilisation after surgery is extremely advantageous. Shorter hospital stay and successful return to pre-admission residence have substantial resource and financial implications for healthcare systems as well as benefits for patients who return to familiar surroundings. Our data suggests the potential reduction in length of hospital stay would be of clinical importance and have the potential for large healthcare savings.

A study strength is the granularity of NHFD observational data, which was lacking in previous observational studies. This includes examining the specific effect of using sedation with spinal anaesthesia; adjusting the analysis for important and clinically relevant variables including fracture classification and pre-operative residence, cognition, and mobility; and assessing important early post-operative outcomes reflecting the quality of acute peri-operative care, including post-operative delirium (including the 4AT subscales) and mobilisation on post-operative day-one. This provided a unique opportunity to examine the effect of anaesthesia on post-operative outcomes in a large population, which captures over 97% of all hip fractures nationwide. Using a nationwide cohort also helps increase the external validity and generalisability of our findings.

A limitation of this work is that causality cannot be inferred from observational data. The reduced risk of delirium in patients receiving spinal anaesthesia without sedation compared with general (22% vs. 28% having 4AT score of 4 or more), suggests an interaction between anaesthesia choice and early delirium following hip fracture surgery. The NHFD data does not collect information regarding why each anaesthetic method was selected, about the specific anaesthetic administered (drugs, dose, depth of sedation), the length of operation and anaesthesia, or the use of specific sedative, opioid or other analgesic agents; factors which may all influence outcomes. We adjusted our models for numerous patient and surgical factors relevant to hip fracture patients including their ASA physical status and admission cognitive status (AMTS); however, we were unable to adjust for other potentially important variables (e.g. frailty and specific medical comorbidities) or unknown confounders.

A research study specifically designed to examine influences on the incidence of delirium would ideally use multiple assessments on each shift of each post-operative day [38]. Any national audit collecting data on 70,000 people a year must be pragmatic and limit the data collection burden on clinical staff, and the NHFD confines its recording of post-operative delirium assessment to the results of a single 4AT in the week following surgery. We recognise that this inevitably means that some episodes of delirium will have been missed from our analysis. Therefore we cannot comment on the severity or duration of delirium in each case, however, this should not affect our comparison of different anaesthetic approaches when exploring the NHFD dataset.

Missing data for some variables may have influenced the findings. We cannot rule out whether patient factors are the link between use of sedation and adverse outcomes (an agitated patient might be more likely to receive sedation and more likely to have adverse outcomes), or anaesthetist factors (anaesthetists who routinely avoid sedation may provide, or work within teams that provide, conditions less likely to result in adverse outcomes). Data from Canada [39, 40], and the inter-unit variation seen within NHFD suggest that patient factors are not as important as hospital and anaesthetist tradition and preference. The Steroids To Reduce the Impact on DElirium (STRIDE) randomised trial compared two levels of propofol sedation in older people having spinal anaesthesia for hip fracture surgery and found no difference overall [5]. However, this did not answer the question of whether avoiding sedation entirely makes a difference. Until formal trials of sedation versus no sedation are performed we are left with suggestive but inconclusive evidence of benefit.

Sedation was not examined in the REGAIN study [18] but common practice was to provide some sedation. The regional with general anaesthesia on postoperative delirium (RAGA-delirium) study avoided sedation in the regional anaesthesia arm. Results suggest no difference in rates of delirium, albeit with very low rates (5-6%) [41]. The iHOPE study does not preclude sedation but does advocate avoidance of deep levels of sedation [22, 23, 35].

The role and outcomes of sedation will require further assessment in ongoing trials, and the future trials recommended by NICE [19]. Our work helps to inform the planning of such studies. However, the effect size that we demonstrate suggests that such studies will need to be very large. A conservative estimate of trial size to demonstrate a reduction in delirium from 30% to 25% is of the order of 1700 participants per group. This means that such an RCT will be challenging to justify and run in the UK, unless it could be embedded within a well-established prospective cohort study [42] that is representative of the general hip fracture population [43].

## Conclusions

Spinal and general anaesthesia may achieve similar outcomes for patients with hip fracture, but within this, it appears that spinal without sedation was associated with improved perioperative outcomes—including a reduced risk of delirium, an increased likelihood of mobilisation by the day after hip fracture surgery, an increased likelihood of returning to admission residence on discharge, and a shorter length of hospital stay. Most of these benefits were not observed in spinal anaesthesia with sedation, suggesting sedation may influence perioperative outcomes in hip fracture surgery. The role and effect of sedation should be assessed in future RCTs of hip fracture patients undergoing spinal anaesthesia.

## Supplementary Information

**Additional file 1: Appendix 1.** Multivariable regression and sensitivity analysis.

**Additional file 2: Table S1.** Baseline characteristics of hip fracture patients by anaesthetic type. **Table S2.** Outcomes of hip fracture patients after surgery by anaesthetic type.

## Data Availability

The study is based on data from the National Hip Fracture Database and was provided within the terms of an NHS Digital sharing agreement. The data do not belong to the authors and may not be shared by the authors, except in aggregate form for publication. Data can be obtained by submitting a research request through the NHS Digital Data Access Request Service.

## Abbreviations

4AT: 4 ‘A’s Test
AMT4: Abbreviated Mental Test 4
AMTS: Abbreviated Mental Test Score
ASA: American Society of Anesthesiologists
CAG: Confidentiality Advisory Group
CI: Confidence interval
GAfREC: Governance Arrangements for Research Ethics Committee
iHOPE: Improve hip fracture outcome in the elderly patient
HRA: Health Research Authority
HQIP: Healthcare Quality Improvement Partnership
IQR: Interquartile range
KPI: Key Performance Indicators
NCAPOP: National Clinical Audit and Patient Outcomes Programme
NHS: National Health Service
NHFD: National Hip Fracture Database
NICE: National Institute for Health and Care Excellence
OR: Odds ratio
ONS: Office for National Statistics
RCT: Randomised controlled trial
RAGA-delirium: Regional with general anaesthesia on postoperative delirium
REGAIN: Regional versus General Anesthesia for Promoting Independence after Hip Fracture
SD: Standard deviation
SMD: Standardised mean difference
STRIDE: Steroids To Reduce the Impact on DElirium
UK: United Kingdom
