# Supplementary material for: The influence of mode of anaesthesia on perioperative outcomes in people with hip fracture: a prospective cohort study from the National Hip Fracture Database for England, Wales and Northern Ireland
Source: BMC Med. 2022 Sep 26;20:319. doi: 10.1186/s12916-022-02517-8 (PMC9511718; doi:10.1186/s12916-022-02517-8)
Supplement: Supplementary file 1 — Additional file 1: Appendix 1. Multivariable regression and sensitivity analysis. [file 12916_2022_2517_MOESM1_ESM.docx]

**Appendix 1: Multivariable regression methods and sensitivity analysis with results**

Multivariable regression models

Multivariable regression models were used to assess the effect of anaesthesia on each individual outcome of interest. All regression models were *a priori* adjusted for the covariates described, with analyses conducted using a complete case analysis. For delirium, analyses were performed using the 4AT score (grouped in the three clinically relevant categories: 0, 1 to 3, and 4 or more), and then separate models were performed for each of the four subscales of the 4AT score, namely alertness, AMT4 (one model compared a score of 1 vs. 0, and another model compared a score of 2 vs. 0), attention, and acute change.

Ordered logistic regression models were used for the delirium 4AT score (p=0.230) and delirium attention (p=0.954) outcomes, with the proportional odds assumptions satisfied using the Brant test of parallel regression assumption. The proportional odds assumption was not met for the delirium AMT4 outcome (Brant test p=0.004), therefore multinomial logistic regression was performed. Logistic regression was used for binary outcome measures, which were delirium alertness, delirium acute change, mobilisation on the day of or day following surgery, whether the patient returned to their admission residence on discharge, and mortality at 30 days following surgery. Linear regression was used to assess the effect of anaesthesia on length of hospital stay.

Sensitivity analysis methods

All analyses were subsequently repeated with patients grouped into the three different anaesthetic types (general anaesthesia, spinal without sedation, or spinal with sedation) in line with NICE recommendations. This grouping also made no distinction regarding the use of nerve blocks, which were subsequently adjusted for. The regression analyses were conducted in identical fashion with the exception that ordered logistic regression was used for the delirium AMT4 outcome (rather than multinomial regression), as the proportional odds assumption was met in this case (Brant test p=0.104).

Sensitivity analysis results

The 4AT score is the total of scores for four subscales: ‘Alertness’, AMT4, ‘Attention’, and ‘Acute change’. In a pre-planned analysis we explored the reasons for these differences between anaesthetic techniques in an examination of these four domains.

When compared with general anaesthesia, spinal without sedation (but not spinal with sedation) was associated with improved ‘Alertness’ (OR=0.88, CI=0.83-0.94; p<0.001). Both spinal approaches were associated with improved AMT4 scores compared with general anaesthesia (without sedation OR=0.91, CI=0.87-0.94, p<0.001; with sedation OR=0.94, CI=0.89-0.98, p=0.008). When compared with general anaesthesia, use of spinal anaesthesia was not associated with any difference in the domains of ‘Attention’ (OR=1.00, CI=0.97-1.03; p=0.969) or ‘Acute change’ (OR=0.99, CI=0.94-1.03; p=0.496), but spinal with sedation was associated with worse ‘Attention’ (OR=1.06, CI=1.01-1.11; p=0.009).
