# Supplementary material for: The influence of mode of anaesthesia on perioperative outcomes in people with hip fracture: a prospective cohort study from the National Hip Fracture Database for England, Wales and Northern Ireland
Source: BMC Med. 2022 Sep 26;20:319. doi: 10.1186/s12916-022-02517-8 (PMC9511718; doi:10.1186/s12916-022-02517-8)
Supplement: Supplementary file 2 — Additional file 2: Table S1. Baseline characteristics of hip fracture patients by anaesthetic type. Table S2. Outcomes of hip fracture patients after surgery by anaesthetic type. [file 12916_2022_2517_MOESM2_ESM.docx]

**Table S1: Baseline characteristics of hip fracture patients by anaesthetic type treated in England, Wales and Northern Ireland during 2018-19, as recorded in the National Hip Fracture Database**

|  |  | Total | | **General anaesthetic** | | **Spinal anaesthetic only** | | **Spinal anaesthetic & sedation** | |
| --- | --- | --- | --- | --- | --- | --- | --- | --- | --- |
|  |  | Number | **%** | Number | **%** | Number | **%** | Number | **%** |
|  |  |  |  |  |  |  |  |  |  |
| **Total** |  | 124,960 | 100 | 68,851 | 55 | 36,982 | 30 | 19,127 | 15 |
| **Year** | 2018 | 61,869 | 50 | 33,603 | 49 | 18,859 | 51 | 9,407 | 49 |
|  | 2019 | 63,091 | 50 | 35,248 | 51 | 18,123 | 49 | 9,720 | 51 |
| **Age (years)** (continuous) |  | Mean=82.7 SD=8.6 | | Mean=82.7 SD=8.7 | | Mean=82.7 SD=8.6 | | Mean=82.5 SD=8.7 | |
| **Age groups** | 60-69 years | 10,937 | 9 | 6,161 | 9 | 3,085 | 8 | 1,691 | 9 |
|  | 70-79 years | 29,250 | 23 | 15,733 | 23 | 8,821 | 24 | 4,696 | 25 |
|  | 80-89 years | 56,469 | 45 | 31,299 | 45 | 16,676 | 45 | 8,494 | 44 |
|  | 90+ years | 28,304 | 23 | 15,658 | 23 | 8,400 | 23 | 4,246 | 22 |
| **Sex** | Female | 88,436 | 71 | 48,807 | 71 | 26,027 | 70 | 13,602 | 71 |
|  | Male | 36,524 | 29 | 20,044 | 29 | 10,955 | 30 | 5,525 | 29 |
| **Fracture type** | Intracapsular | 72,704 | 58 | 38,562 | 56 | 22,298 | 60 | 11,844 | 62 |
|  | Extracapsular, including other | 52,137 | 42 | 30,196 | 44 | 14,668 | 40 | 7,273 | 38 |
|  | Missing | 119 | <1 | 93 | <1 | 16 | <1 | 10 | <1 |
| **Pathology** | No malignancy | 119,033 | 95 | 65,473 | 95 | 35,298 | 95 | 18,262 | 95 |
|  | Malignancy present | 1,478 | 1 | 833 | 1 | 429 | 1 | 216 | 1 |
|  | Missing | 4,449 | 4 | 2,545 | 4 | 1,255 | 3 | 649 | 3 |
| **Region** | East Midlands | 8,808 | 7 | 4,241 | 6 | 3,793 | 10 | 774 | 4 |
|  | East of England | 13,887 | 11 | 8,777 | 13 | 4,405 | 12 | 705 | 4 |
|  | London | 10,926 | 9 | 6,379 | 9 | 2,956 | 8 | 1,591 | 8 |
|  | North East | 6,887 | 6 | 3,403 | 5 | 1,979 | 5 | 1,505 | 8 |
|  | North West | 15,897 | 13 | 9,045 | 13 | 5,158 | 14 | 1,694 | 9 |
|  | Northern Ireland | 4,009 | 3 | 1,322 | 2 | 648 | 2 | 2,039 | 11 |
|  | South Central | 8,101 | 6 | 4,885 | 7 | 1,529 | 4 | 1,687 | 9 |
|  | South East | 10,812 | 9 | 4,588 | 7 | 2,923 | 8 | 3,301 | 17 |
|  | South West | 14,058 | 11 | 8,683 | 13 | 2,619 | 7 | 2,756 | 14 |
|  | Wales | 7,691 | 6 | 4,657 | 7 | 2,360 | 6 | 674 | 4 |
|  | West Midlands | 12,076 | 10 | 7,603 | 11 | 3,372 | 9 | 1,101 | 6 |
|  | Yorkshire & the Humber | 11,808 | 9 | 5,268 | 8 | 5,240 | 14 | 1,300 | 7 |
| **Admission source** | Own home / sheltered housing | 102,292 | 82 | 54,868 | 80 | 31,439 | 85 | 15,985 | 84 |
|  | Nursing care | 8,994 | 7 | 5,457 | 8 | 2,122 | 6 | 1,415 | 7 |
|  | Residential care | 13,611 | 11 | 8,488 | 12 | 3,404 | 9 | 1,719 | 9 |
|  | Missing | 63 | <1 | 38 | <1 | 17 | <1 | 8 | <1 |
| **ASA grade** | ASA grade 1 / 2 | 28,940 | 23 | 14,961 | 22 | 8,840 | 24 | 5,139 | 27 |
|  | ASA grade 3 | 71,734 | 57 | 39,666 | 58 | 21,192 | 57 | 10,876 | 57 |
|  | ASA grade 4 /5 | 22,341 | 18 | 12,979 | 19 | 6,424 | 17 | 2,938 | 15 |
|  | Missing | 1,945 | 2 | 1,245 | 2 | 526 | 1 | 174 | 1 |
| **Preinjury mobility** | Freely mobile without aids | 45,388 | 36 | 23,970 | 35 | 13,970 | 38 | 7,448 | 39 |
|  | Mobile outdoors with 1 aid, or 2 aids or frame | 46,228 | 37 | 25,326 | 37 | 14,115 | 38 | 6,787 | 35 |
|  | Some indoor mobility but never goes out, or no functional mobility | 32,207 | 26 | 18,827 | 27 | 8,630 | 23 | 4,750 | 25 |
|  | Missing | 1,137 | 1 | 728 | 1 | 267 | 1 | 142 | 1 |
| **Pre-operative cognitive state (Abbreviated mental test score)** | 0-7 | 43,690 | 35 | 26,202 | 38 | 11,515 | 31 | 5,973 | 31 |
|  | 8-10* | 75,834 | 61 | 39,738 | 58 | 24,055 | 65 | 12,041 | 63 |
|  | Missing | 5,436 | 4 | 2,911 | 4 | 1,412 | 4 | 1,113 | 6 |
| **Operation type** | Hemiarthroplasty | 55,176 | 44 | 29,661 | 43 | 16,721 | 45 | 8,794 | 46 |
|  | Total Hip Replacement | 10,030 | 8 | 4,709 | 7 | 3,154 | 9 | 2,167 | 11 |
|  | Internal fixation - cannulated screws | 3,291 | 3 | 1,817 | 3 | 1,078 | 3 | 396 | 2 |
|  | Internal fixation - intramedullary nail | 17,783 | 14 | 10,929 | 16 | 4,423 | 12 | 2,431 | 13 |
|  | Internal fixation - sliding hip screw | 38,402 | 31 | 21,564 | 31 | 11,535 | 31 | 5,303 | 28 |
|  | Other / missing | 278 | <1 | 171 | <1 | 71 | <1 | 36 | <1 |
| **Grade of surgeon** | Consultant | 87,934 | 70 | 48,032 | 70 | 26,067 | 70 | 13,835 | 72 |
|  | Other | 36,714 | 29 | 20,617 | 30 | 10,828 | 29 | 5,269 | 28 |
|  | Missing | 312 | <1 | 202 | <1 | 87 | <1 | 23 | <1 |
| **Grade of anaesthetist present at operation** | Consultant | 106,803 | 85 | 59,107 | 86 | 31,234 | 84 | 16,462 | 86 |
|  | Other | 16,712 | 13 | 8,850 | 13 | 5,330 | 14 | 2,533 | 13 |
|  | Missing | 1,444 | 1 | 894 | 1 | 418 | 1 | 132 | 1 |
| **Time to theatre from admission (hours)** |  | Median=24.7 IQR=18.7-40.6 | | Median=24.7 IQR=18.7-40.4 | | Median=24.6 IQR=18.7-40.3 | | Median=24.8 IQR=18.8-40.2 | |
| **Time to theatre from admission >=36 hours** | Yes | 37,908 | 30 | 20,734 | 30 | 11,069 | 30 | 6,105 | 32 |
|  | No | 87,052 | 70 | 48,117 | 70 | 25,913 | 70 | 13,022 | 68 |
| **Nerve block in A&E or the ward before arrival in theatre suite** | Yes | 60,848 | 49 | 32,713 | 48 | 18,031 | 49 | 10,104 | 53 |
|  | No | 58,930 | 47 | 33,295 | 48 | 16,825 | 45 | 8,810 | 46 |
|  | Missing | 5,182 | 4 | 2,843 | 4 | 2,126 | 6 | 213 | 1 |

**Table S2: Outcomes of hip fracture patients after surgery by anaesthetic type for those treated in England, Wales and Northern Ireland during 2018-19, as recorded in the National Hip Fracture Database**

|  |  | **Total** | | **General anaesthetic** | | **Spinal anaesthetic only** | | **Spinal anaesthetic & sedation** | |
| --- | --- | --- | --- | --- | --- | --- | --- | --- | --- |
|  |  | Number | **%** | Number | **%** | Number | **%** | Number | **%** |
|  |  |  |  |  |  |  |  |  |  |
| **Total** |  | 124,960 | 100 | 68,851 | 55 | 36,982 | 30 | 19,127 | 15 |
| **Delirium assessment (categorical)** | 0 - Delirium or cognitive impairment unlikely | 55,410 | 44 | 28,888 | 42 | 17,457 | 47 | 9,065 | 47 |
|  | 1-3 Possible cognitive impairment | 27,232 | 22 | 15,055 | 22 | 8,237 | 22 | 3,940 | 21 |
|  | 4+ Possible delirium or cognitive impairment | 32,244 | 26 | 19,036 | 28 | 8,255 | 22 | 4,953 | 26 |
|  | Missing | 10,074 | 8 | 5,872 | 9 | 3,033 | 8 | 1,169 | 6 |
| **Delirium Alertness** | 0 | 100,452 | 80 | 54,490 | 79 | 29,946 | 81 | 16,016 | 84 |
|  | 4 | 7,029 | 6 | 4,294 | 6 | 1,757 | 5 | 978 | 5 |
|  | Missing | 17,479 | 14 | 10,067 | 15 | 5,279 | 14 | 2,133 | 11 |
| **Delirium AMT4** | 0 | 62,233 | 50 | 32,351 | 47 | 19,461 | 53 | 10,421 | 54 |
|  | 1 | 13,188 | 11 | 7,110 | 10 | 4,064 | 11 | 2,014 | 11 |
|  | 2 | 32,119 | 26 | 19,363 | 28 | 8,194 | 22 | 4,562 | 24 |
|  | Missing | 17,420 | 14 | 10,027 | 15 | 5,263 | 14 | 2,130 | 11 |
| **Delirium Attention** | 0 | 61,788 | 49 | 32,290 | 47 | 19,279 | 52 | 10,219 | 53 |
|  | 1 | 21,361 | 17 | 11,978 | 17 | 6,209 | 17 | 3,174 | 17 |
|  | 2 | 24,386 | 20 | 14,558 | 21 | 6,221 | 17 | 3,607 | 19 |
|  | Missing | 17,425 | 14 | 10,025 | 15 | 5,273 | 14 | 2,127 | 11 |
| **Delirium Acute change** | 0 | 95,723 | 77 | 51,978 | 75 | 28,540 | 77 | 15,183 | 79 |
|  | 4 | 11,755 | 9 | 6,795 | 10 | 3,155 | 9 | 1,805 | 9 |
|  | Missing | 17,504 | 14 | 10,078 | 15 | 5,287 | 14 | 2,139 | 11 |
| **Mobilised on day of or day following surgery** | No | 24,752 | 20 | 14,071 | 20 | 7,043 | 19 | 3,638 | 19 |
|  | Yes | 99,772 | 80 | 54,502 | 79 | 29,817 | 81 | 15,453 | 81 |
|  | Missing | 436 | <1 | 278 | <1 | 122 | <1 | 36 | 0 |
| **Length of hospital stay (days)** (continuous) |  | Median=15  IQR=9-25 | | Median=15 IQR=9-26 | | Median=15  IQR=9-25 | | Median=14 IQR=9-23 | |
| **Returned to admission source** | Yes | 81,060 | 65 | 44,128 | 64 | 24,411 | 66 | 12,521 | 65 |
|  | No | 42,463 | 34 | 23,918 | 35 | 12,190 | 33 | 6,355 | 33 |
|  | Missing | 1,437 | 1 | 805 | 1 | 381 | 1 | 251 | 1 |
| **Life status at 30 days** | Alive | 117,998 | 94 | 64,824 | 94 | 34,910 | 94 | 18,264 | 95 |
|  | Dead | 6,962 | 6 | 4,027 | 6 | 2,072 | 6 | 863 | 5 |
